# Efficacy and safety of surufatinib in the treatment of thymic neuroendocrine tumors: a 102-case retrospective study.

**DOI:** 10.1093/oncolo/oyaf072

**Published:** 2025-05-27

**Authors:** Xuebing Jia, Zhichen Sun, Junyan Xu, Dan Huang, Fengzhen Chen, Jie Chen, Yun Liang

**Affiliations:** Centre for Neuroendocrine Tumors, Fudan University Shanghai Cancer Center, Shanghai 200032, People’s Republic of China; Centre for Neuroendocrine Tumors, Fudan University Shanghai Cancer Center, Shanghai 200032, People’s Republic of China; Department of Nuclear Medicine, Fudan University Shanghai Cancer Center, Shanghai 200032, People’s Republic of China; Department of Pathology, Fudan University Shanghai Cancer Center, Shanghai 200032, People’s Republic of China; Centre for Neuroendocrine Tumors, Fudan University Shanghai Cancer Center, Shanghai 200032, People’s Republic of China; Centre for Neuroendocrine Tumors, Fudan University Shanghai Cancer Center, Shanghai 200032, People’s Republic of China; Department of Oncology, Shanghai Medical College, Fudan University, Shanghai 200032, People’s Republic of China; Centre for Neuroendocrine Tumors, Fudan University Shanghai Cancer Center, Shanghai 200032, People’s Republic of China; Department of Oncology, Shanghai Medical College, Fudan University, Shanghai 200032, People’s Republic of China

**Keywords:** neuroendocrine tumor, thymic, surufatinib, treatment

## Abstract

**Introduction or hypothesis:**

The SANET-ep study confirmed that patients with non-pancreatic NET could benefit from surufatinib treatment compared with placebo. However, there is a lack of sufficient retrospective data on surufatinib’s efficacy in treating thymic neuroendocrine tumors (TNETs). This study aimed to evaluate the efficacy and safety of surufatinib in well-differentiated TNETs by conducting a single center retrospective analysis.

**Methods:**

We conducted a retrospective study at Fudan University Shanghai Cancer Center, including 102 patients with TNETs. This study assessed the efficacy and safety of surufatinib in this population.

**Results:**

Median progression-free survival was 10.0 months, ORR was 5.6%, and DCR was 83.1%. Surufatinib performed better in first-line treatment compared to secondary treatment in terms of ORR, DCR, and mPFS. The most common adverse events were diarrhea (15.7%), hypertension (19.1%), proteinuria (13.5%), and hypothyroidism (5.6%). About 33.3% of patients required dosage reduction from 300 mg/day to 200 mg/day (or 250 mg/day) due to adverse events, which in most cases were mitigated or disappeared with dose reduction.

**Conclusions:**

For patients with advanced, progressive, well-differentiated TNETs, Surufatinib can be used as an option with a high mPFS and DCR. Moreover, the safety profile of surufatinib was found to be acceptable. These findings suggest that surufatinib could potentially serve as an innovative therapeutic option for this particular patient population.

**Implications for practice**

In summary, surufatinib demonstrated a significant and meaningful enhancement in PFS for patients with advanced, progressive, well-differentiated TNETs, irrespective of their previous antitumor therapy and specific extra-pancreatic origin. Moreover, the safety profile of surufatinib was found to be acceptable. These findings suggest that surufatinib could potentially serve as an innovative therapeutic option for this particular patient population.

## Introduction

Neuroendocrine neoplasms (NENs), a diverse group of rare tumors originating from neuroendocrine cells, have gained increased attention with advances in diagnostic techniques. According to the Surveillance, Epidemiology and End Results (SEER) database, thymic neuroendocrine tumors (TNETs) account for less than 0.5% of all neuroendocrine neoplasms and approximately 5% of thymic tumor.^1^ TNETs, known for their aggressive nature and relatively poor prognosis compared to other neuroendocrine tumors, are frequently associated with ectopic adrenocorticotropic hormone (ACTH) syndrome and multiple endocrine neoplasia (MEN).^2-5^ Due to the low expression of somatostatin receptors (SSTR), the utilization of somatostatin analogs (SSA) and peptide receptor radionuclide therapy (PRRT) in TNETs is limited, leading to diminished therapeutic outcomes compared to gastrointestinal pancreatic neuroendocrine tumors.^6,7^ At diagnosis, many patients present with metastatic disease, necessitating a focus on symptom management and tumor progression control. In recent years, targeted therapies have attracted significant interest for managing advanced TNETs that are unsuitable for surgery. Surufatinib, an orally administered small molecule tyrosine kinase inhibitor (TKI), targets vascular endothelial growth factor receptors (VEGFR) 1, 2, and 3, fibroblast growth factor receptor type 1 (FGFR1), and colony stimulating factor-1 receptor (CSF-1R).^8^ This mechanism enables surufatinib to both inhibit angiogenesis via VEGFR and FGFR1. However, the evidence for immune activation is currently limited.

Clinical trials showed promising antitumor efficacy for Surufatinib with manageable toxicity in patients with advanced neuroendocrine tumors (NETs) in phase I and phase Ib/II trials.^9,10^ Following these findings, Xu et al^11^ initiated the SANET-ep study, a randomized, double-blind, phase III clinical trial comparing surufatinib with placebo in 198 patients with advanced, well-differentiated extra-pancreatic NETs. This study revealed that surufatinib prolonged progression-free survival (PFS) in non-pancreatic well to moderately differentiated NET patients, increasing from 3.8 months in the control group to 9.2 months in the treatment group. Moreover, it resulted in an objective response rate (ORR) of 10.3% and a disease control rate (DCR) of 86.5%. The most common grade 3 or higher adverse events were hypertension (36%) and proteinuria (19%). Based on the SANET-ep study, surufatinib was approved in China for the treatment of late-stage, well-differentiated, extra-pancreatic NET [5, 6].

However, while the clinical trials have demonstrated surufatinib’s efficacy in extra-pancreatic NET, there is still a need for large-scale, real-world evidence to validate its clinical application, efficacy, and safety in treating TNETs. To address this gap, we conducted a retrospective study to assess the efficacy and safety of surufatinib in well-differentiated TNETs at Fudan University Shanghai Cancer Center, China. A total of 102 patients with TNETs were included in this evaluation.

## Materials and methods

### Patient

A total of 102 patients with well-differentiated TNETs (either typical carcinoid, TC, or atypical carcinoid, AC) who received surufatinib treatment at Fudan University Shanghai Cancer Center, China, between June 2021 and June 2024, were included in this study. The aim was to investigate the efficacy and safety of surufatinib in Chinese TNETs patients. Exclusion criteria included neuroendocrine carcinoma (NEC), non-neuroendocrine histological component and patients who did not receive surufatinib. Data were retrospectively collected for all eligible patients, including basic information and treatment protocols, through the electronic medical record system. Clinical characteristics of the 102 patients treated with surufatinib are summarized in **Table 1**. Functional TNETs were defined as tumors that secreted hormone causing clinical symptoms. Pathological diagnoses were made in accordance with the 2021 World Health Organization (WHO) classification of tumors. The tumor-node-metastasis stage was determined using the American Joint Committee on Cancer (AJCC) staging system. This study was approved by the Institutional Review Board of Fudan University Shanghai Cancer Center (Number: 050432-4-2108).

**Table 1.** Clinical characteristics of 102 patients with thymic neuroendocrine tumors treated with surufatinib.

Clinical characteristics	*n*	%
Sex		
Male	71	69.6
Female	31	30.4
Median age, years (range)	51 (22-81)	
Functional status		
Nonfunctional	90	88.2
Functional	12	11.8
Median Ki-67 index, % (range)	16 (1-45)	
Somatostatin receptor expression		
Negative	78	76.5
Positive	18	17.6
Unknown	6	5.9
Tumor grade		
TC	32	31.4
AC	70	68.6
Median primary focal size, cm (range)	6.9 (0.5-20)	
Sites of metastases		
Lymph node	79	77.5
Bone	68	66.7
Pleura	43	42.2
Lung	34	33.3
Pericardium	20	19.6
Pancrea	19	18.6
Liver	16	15.7
Multiple endocrine neoplasia type 1		
Yes	13	12.7
No	89	87.3
Treatment lines of surufatinib		
1	38	37.3
2	41	40.2
3	10	9.8
≥4	13	12.7
Dosages of surufatinib (mg/day)		
200	72	70.6
250	8	7.8
300	22	21.6
Combined therapy		
Monotherapy	89	87.3
With somatostatin analogs	2	2.0
With interventional therapy	3	2.9
With immunological therapy	8	7.8
Previous therapy		
Surgical resection of primary focus		
Yes	62	60.8
No	40	39.2
Radiation therapy		
Yes	37	36.3
No	65	63.7

### Treatment strategy

Patients received surufatinib orally once daily, with an initial dose of 200 mg/day, 250 mg/day or 300 mg/day, depending on the individual’s clinical condition. If the patient was unable to tolerate the 300 mg/day dose, the dosage was reduced to 200 mg/day or 250 mg/day based on the severity of adverse events and the overall patient assessment. The treatment continued until either tumor progression occurred or the patient was unable to tolerate the drug due to adverse effects. While the treatment strategy and assessment methods were based on standardized clinical practices during the treatment period, the study design itself was retrospective.

### Efficacy and adverse event assessment

PFS was measured from the start of surufatinib until disease progression or cancer-related death. ORR was defined as the percentage of patients achieving complete remission (CR) or partial response (PR), while DCR was calculated by combining CR, PR, and stable disease (SD). The patients’ clinical pathological data, their signs and symptoms, and laboratory test and imaging data during and after surufatinib administration were collected and analyzed retrospectively. All adverse events (AEs) were recorded according to the Common Terminology Criteria for Adverse Events (CTCAE, version 5.0), with the time of AE occurrence defined as the time from the start of surufatinib until the first recorded AE.

### Statistical analysis

Data analyses and visualizations were performed using IBM SPSS Statistics for Windows, version 27.0 (IBMCorp.). Clinical and demographic data, adverse events, and efficacy results were summarized using descriptive statistics, with general characteristics presented as percentages, medians, and ranges. Univariate analyses were conducted using the log-rank test, and variables with a *P*-values less than.2 were included in a Cox regression model for multivariate analysis. Hazard ratios (HRs) were calculated and reported with a corresponding 95% confidence interval (CI). ORR, DCR, and SD rates were also summarized with 95% CI calculated. Differences between variables are considered statistically significant at *P*- values < .05.

## Results

### Clinic‐pathological characteristics

A total of 102 TNETs patients treated with surufatinib were enrolled in this study. The baseline clinicopathological characteristics are summarized in Table 1. Of the 102 patients, 71 were male (69.6%) and 31 were female (30.4%), with a median age of 51 years (range 22-81). Most patients had non-functioning TNET (*n* = 90, 88.2%), while 12 patients (11.8%) had functional TNETs. The median Ki-67 index was 16% (range 1-45%). According to the Krenning scale, defining lesions with uptake ≤liver as negative/weak and >liver as positive. Among the patients tested for SSTR expression, 78 patients (76.5%) had negative expression, and only 18 (17.6%) patients had positive expression. 32 patients (31.4%) were diagnosed with TC, while 70 (68.6%) were AC. The median tumor size was 6.9 cm (range 0.5-20 cm). Metastatic sites included lymph nodes (79, 77.5%), bone (68, 66.7%), pleura (43, 42.2%), lung (34, 33.3%), pericardium (20,19.6%), pancreas (19,18.6%) and liver (16,15.7%). 13 patients (12.7%) had MEN1 syndrome.

All patients received surufatinib, with 70.6% receiving 200 mg/day, 7.8% receiving 250 mg/day, and 21.6% receiving 300 mg/day, as reported in Table 1. Most patients (79, 77.5%) received surufatinib as first or second‐line therapy. The majority of patients (89, 87.3%) received surufatinib as monotherapy, while 2 (2%) received a combination with SSAs, 3 (2.9%) received interventional therapy (transarterial embolization for liver metastases), and 8 (7.8%) received combination with immunotherapy. Prior to surufatinib treatment, 62 patients (60.8%) underwent surgical resection of the primary tumor, and 37 patients (36.3%) received radiation therapy.

**Figure 1.**
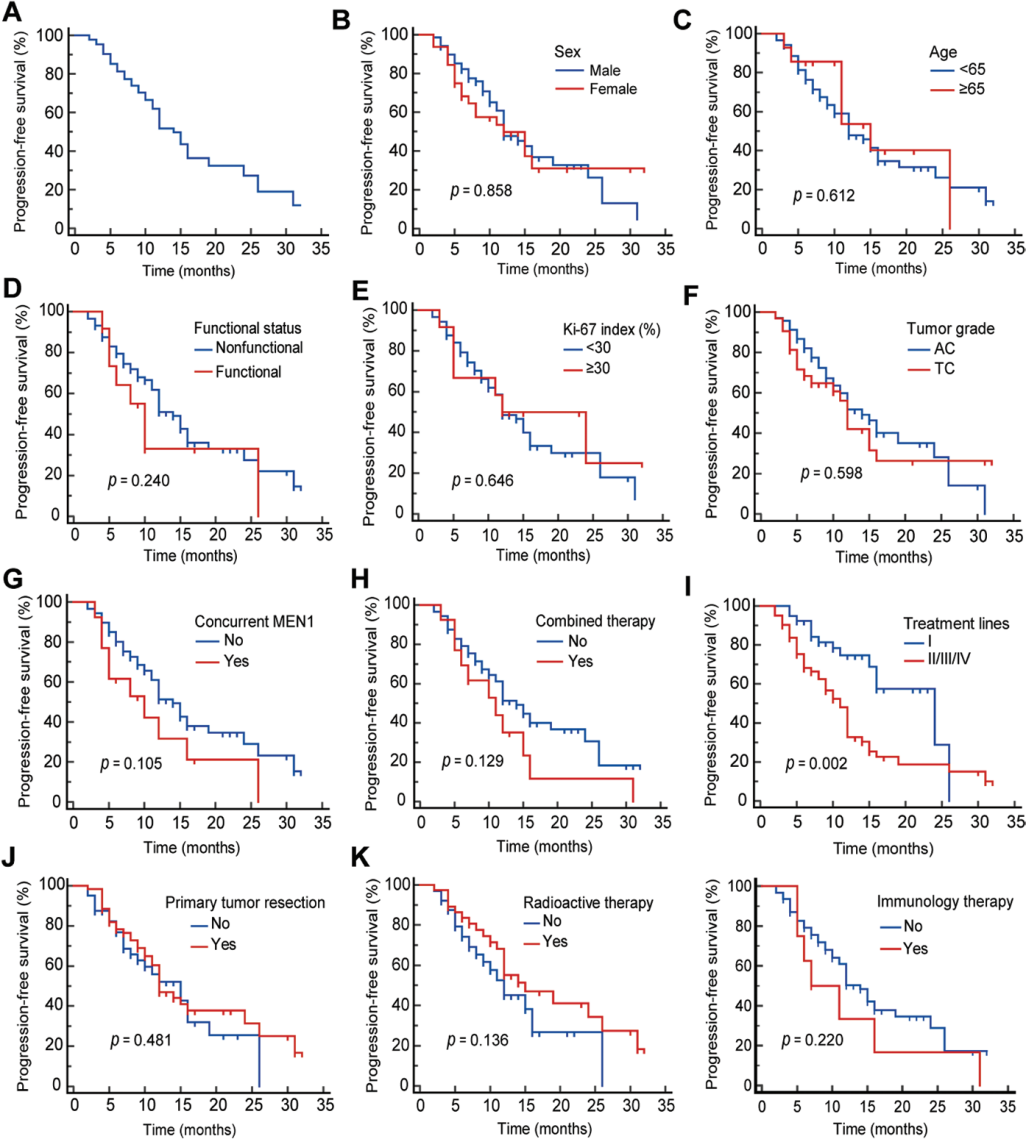
Curve of progression-free survival analysis of 102 TNET patients and univariate analysis of progression-free survival. (A) Curve of progression-free survival analysis of 102 TNET patients. (B) Sex. (C) Age. (D) Functional status. (E) Ki-67 index. (F) Tumor grade. (G) Concurrent MEN. (H) Combined therapy. (I) Treatment lines. (J) Primary tumor resection. (K) Radiation therapy. (L) Immunology therapy

Multivariate analysis revealed that patients receiving surufatinib as first-line therapy had significantly better PFS compared to those receiving it as a back-line treatment. Additionally, patients who underwent radiotherapy prior to surufatinib also had better PFS than those who did not. There were no significant associations between PFS and factors such as gender, age, tumor functional status, Ki-67 index, tumor grade, presence of MEN1 syndrome, use of other anti-tumor treatments or previous surgical intervention before surufatinib treatment (***P*** = 0.858, ***P*** = 0.612, ***P*** = 0.240, ***P*** = 0.646, ***P*** = 0.598, ***P*** = 0.105, ***P*** = 0.133, ***P*** = 0.481), as shown in Table 2 and Figure 1.

**Table 2.** Univariate and multivariate analysis of factors associated with progression-free survival in 102 TNET patients.

Variables	Univariate analysis			Multivariate analysis		
	HR	95% CI	*P* value	HR	95% CI	*P* value
Sex (female vs. male)	1.055	0.589-1.890	0.858			
Age, years (≥65 vs. < 65)	0.821	0.383-1.759	0.612			
Functional states (func vs. non)	1.730	0.694-4.314	0.240			
Ki-67 index, % (≥30 vs. < 30)	0.835	0.386-1.804	0.646			
Tumor grade (TC vs. AC)	1.171	0.651-2.107	0.598			
MEN1 (yes vs. no)	2.033	0.856-4.749	0.105	1.736	0.872-3.458	0.117
Combined therapy (yes vs. no)	1.852	0.862-4.796	0.133	1.219	0.623-2.383	0.563
Treatment lines (II/III/IV vs. I)	2.377	1.371-4.120	0.002	3.384	1.752-6.537	<0.005
Primary tumor resection (yes vs. no)	0.816	0.464-1.435	0.481			
Radiation therapy (yes vs. no)	0.655	0.375-1.143	0.136	0.425	0.228-0.793	0.007
Immunology therapy (yes vs. no)	1.867	0.689-5.060	0.220			

Abbreviations: AC, atypical carcinoid; CI, confidence interval; HR, hazard ratio; MEN1, multiple endocrine neoplasia type 1; TC, typical carcinoid.

### Efficacy evaluation

To exclude the influence of combination therapy, we conducted an efficacy analysis on 89 patients who received surufatinib as a monotherapy. The follow-up period ranged from 2 to 36 months, with a median follow-up of 16.5 months. At the end of the follow-up, 6 patients had died due to progressive disease (PD), while 32 patients were still undergoing surufatinib treatment. Imaging assessments showed that 5 patients achieved PR, and 69 achieved SD, resulting in an SD rate of 77.5%, an ORR of 5.6%, and a DCR of 83.1%, and the median PFS (mPFS) of 10.0 months. The ORR for surufatinib as first-line, second-line, and third-line or higher treatment was respectively observed at rates of 8.6%, 5.9%, and 0.0%. The DCR for surufatinib as first-line, second-line, and third-line or higher treatment was respectively found at rates of 100%,76.5%, and 65.0%. The mPFS for surufatinib as first-line, second-line, and third-line or higher treatment was respectively determined at rates of 12.0 months, 9.0 months, and 7.0 months (Table 3). DCRs were 81.8%,100%, and 83.3% at doses of 200 mg, 250 mg, and 300 mg, respectively, while mPFS for those doses were 9.0 months, 14.0 months, and 11.0 months (Table 4).

**Table 3.** Clinical efficacy of 89 patients who received different lines of surufatinib.

Evaluation result	Total (*N* = 89)	Lines of surufatinib treatment		
		First line (*N* = 35)	Second line (*N* = 34)	Third lines and above (*N* = 20)
PR, % (*n*/*N*)	5.6 (5/89)	8.6 (3/35)	5.9 (2/34)	0.0 (0/20)
SD, % (*n*/*N*)	77.5 (69/89)	91.4 (32/35)	70.6 (24/34)	65.0 (13/20)
^PD, %^ (*n*/*N*)	16.9 (15/89)	0.0 (0/35)	23.5 (8/34)	35.0 (7/20)
ORR, %	5.6	8.6	5.9	0.0
DCR, %	83.1	100.0	76.5	65.0
mPFS, months	10.0	12.0	9.0	7.0

Abbreviations: DCR, disease control rate; mPFS, median progression free survival; ORR, overall response rate; PD, progressive disease; PR, partial remission; SD, stable disease.

**Table 4.** Clinical efficacy of 89 patients who received different dosages of surufatinib.

Evaluation result	Total (*N* = 89)	Dosages of surufatinib (mg/day)		
		200 (*N* = 66)	250 (*N* = 5)	300 (*N* = 18)
PR, % (*n*/*N*)	5.6 (5/89)	7.6 (5/66)	0.0 (0/5)	0.0 (0/18)
SD, % (*n*/*N*)	77.5 (69/89)	74.2 (49/66)	100.0 (5/5)	83.3 (15/18)
PD, % (*n*/*N*)	16.9 (15/89)	18.2 (12/66)	0.0 (0/5)	16.7 (3/18)
ORR, %	5.6	7.6	0.0	0.0
DCR, %	83.1	81.8	100.0	83.3
mPFS, months	10.0	9.0	14.0	11.0

Abbreviations: DCR, disease control rate, mPFS, median progression free survival, ORR, overall response rate, PD, progressive disease, PR, partial remission, SD, stable disease.

### Adverse events

Among the 89 patients treated with surufatinib as a monotherapy, AEs occurred in 43.8% (39/89) of patients. The most common AEs were hypertension (17, 19.1%), diarrhea (14, 15.7%), proteinuria (12, 13.5%), and hypothyroidism (5, 5.6%). A total of 10 patients experienced grade 3 or higher AEs, primarily grade 3 or higher hypertension (5 cases), grade 3 or higher diarrhea (4 cases), and grade 3 or higher gastrointestinal reaction (1 case). These patients temporarily discontinued medication and resumed treatment at a reduced dosage level only after their adverse reactions had subsided to grade 0-1.

In the 66 patients who received 200 mg/day, the AE incidence was 36.4% (24/66). The most common treatment-related AEs were hypertension (15.2%), proteinuria (10.6%), hypothyroidism (6.1%), and diarrhea (6.1%). Three patients (4.5%) experienced grade 3 or higher AEs, all involving grade 3 or higher hypertension. For the 3 patients who developed grade 3 or higher adverse events while taking 200 mg/d, medication was permanently discontinued. Among the 5 patients receiving 250mg/day, the AEs incidence was 60% (3/5), with diarrhea (40%), hypertension (40%), and proteinuria (20%). Three patients (60%) experienced grade 3 or higher AEs including 2 cases of grade 3 or higher diarrhea (40%) and 2 cases of grade 3 or higher hypertension (40%). Among the 18 patients receiving 300 mg/day, the AE incidence was 61.1% (11/18), with diarrhea (44.4%), hypertension (27.8%), and proteinuria (22.2%), while 1 patient developed hypothyroidism (5.6%). Six patients (33.3%) experienced grade-3 or higher AE including 4 cases of grade 3 or higher diarrhea (22.2%), 1 case of grade 3 or higher hypertension (5.6%), and 1 case of severe gastrointestinal reaction (5.6%). Among 18 patients on 300 mg/day, 33.3% (6/18) required a dose reduction to 200 mg/day or 250 mg/day due to intolerance, with subsequent symptom improvement. Similarly, among 5 patients on 250 mg/day, 60% (3/5) required a reduction to 200 mg/day, also leading to symptom improvement.

No treatment-related deaths were reported. AEs occurred within 1 to 52 weeks of surufatinib initiation, as shown in Table 5, which details the time range and severity of events. Transient hypertension was observed but was effectively managed with antihypertensive medication. Of the 39 patients who experienced AEs, 3 achieved PR while 30 maintained SD. The mPFS for these individuals was recorded at 10.0 months, aligning closely with the overall mPFS (10.0 months) observed across all 89 patients.

**Table 5.** Principal adverse events (AEs) in 89 patients who received varying dosages of surufatinib.

Adverse events (grade)	Range of AE occurrence time	Dosages of surufatinib (mg/day), *n* (%)			Total (N = 89)
		200 (*N* = 66)	250 (*N* = 5)	300 (*N* = 18)	
Hypothyroidism					
Any Gr	Weeks 8-2	4 (6.1)	0 (0.0)	1 (5.6)	5 (5.6)
CTCAE ≥Gr 3		0 (0.0)	0 (0.0)	0 (0.0)	0 (0.0)
Proteinuria					
Any Gr	Weeks 2-32	7 (10.6)	1 (20.0)	4 (22.2)	12 (13.5)
CTCAE ≥Gr 3		0 (0.0)	0 (0.0)	0 (0.0)	0 (0.0)
Diarrhea					
Any Gr	Weeks 1-52	4 (6.1)	2 (40.0)	8 (44.4)	14 (15.7)
CTCAE ≥Gr 3	Weeks 4-52	0 (0.0)	2 (40.0)	4 (22.2)	4 (4.5)
Hypertension					
Any Gr	Weeks 2-44	10 (15.2)	2 (40.0)	5 (27.8)	17 (19.1)
CTCAE ≥Gr 3	Weeks 2-14	3 (4.5)	1 (20.0)	1 (5.6)	5 (5.6)
Others					
Any Gr	Weeks 1-10	3 (4.5)	0 (0.0)	3 (16.7)	6 (6.7)
CTCAE ≥ Gr 3	Weeks 10	0 (0.0)	0 (0.0)	1 (5.6)	1 (1.1)

## Discussion

In this retrospective, single-center study, we investigated real-world clinical practice for 102 patients with TNETs treated with surufatinib. According to the literature, functional TNETs account for about 12%, with ACTH-secreting and carcinoid syndrome being the most common types, while Cushing syndrome accounts for 11.8%.^2,12,13^ Similarly, we found that 11.8% of TNETs were functional tumors, but only 5 cases of functional TNETs with ACTH-secreting, accounted for 4.9%. According to reports, approximately 70% of TNETs exhibit a lack of SSTR expression.^14-16^ In our study, we observed that 76.5% of patients demonstrated negative expression of SSTRs, which imposes limitations on the utilization of SSA and PRRT and may consequently diminish therapeutic efficacy in comparison to gastrointestinal pancreatic neuroendocrine tumors. The majority of TNETs patients (77.5%) had metastasis in the lymph nodes, while 66.7% had bone metastasis. Lymph node metastasis can be easily detected on CT scans, but careful evaluation is needed for detecting bone metastasis, especially with whole-body bone window scanning. Unlike neuroendocrine tumors of the digestive tract, liver metastasis is relatively rare in TNETs patients (15.7%), but there is still a risk, so abdominal and pelvic examinations are recommended as well.

In our single-center retrospective study of 89 Chinese TNET patients, surufatinib treatment resulted in a median PFS of 10.0 months, with an ORR of 5.6% and a DCR of 83.1%. The SANET-ep study, the first phase III clinical trial specifically targeting Chinese NET patients, enrolled 198 individuals with advanced non-pancreatic NETs. Compared to placebo, surufatinib significantly extended the median PFS in these patients from 3.8 months to 9.2 months. Furthermore, patients receiving surufatinib showed an objective response rate (ORR) of 10.3% and a disease control rate (DCR) of 86.5%. However, only 18 TNETs were included in this study. Our findings indicated a slightly longer PFS but a lower ORR compared to the SANET-ep phase III clinical trial, and this discrepancy may be attributed to site-specific factors. For metastatic lung and thymic NETs (excluding small cell lung cancer), there are relatively few studies assessing the efficacy of TKIs. A single-arm phase II trial evaluated pazopanib in 44 patients (including 5 with lung NETs and 3 with thymic NETs), revealing a median PFS of only 3.4 months for patients with lung/thymic NETs, which was significantly lower than that of patients with pancreatic or GI NETs (*P* = 0.005).^17^ The phase III clinical trial of CABINET demonstrated that cabozantinib had a median progression-free survival of 8.4 months in a cohort of 203 patients with extra-pancreatic neuroendocrine tumors, with the ORR of 5%,^18,19^ while there were only 6 TNET patients included in this study. However, the PFS of both surufatinib and previous TKIs in epNETs was limited to within one year, which could be attributed to the inherent limitations of anti-vascular TKIs or possibly due to the lack of blood supply characteristics of epNET.

In our study, the majority of enrolled patients (88.2%) had non-functional tumors, which aligns with the clinical and pathological characteristics of Chinese TNET patients observed in epidemiological studies.^6,20^ Subgroup analysis showed that the functional status did not impact PFS, suggesting that surufatinib may be effective regardless of tumor functionality. Furthermore, no significant association was found between PFS and factors such as gender, age, Ki-67 index, tumor type, or the presence of MEN1 syndrome, as depicted in Table 2. Moreover, undergoing surgery before initiating surufatinib did not have a substantial impact on PFS (*P* = 0.481), suggesting that the efficacy of surufatinib is independent of primary tumor surgical intervention. Additionally, combination therapy involving surufatinib and other treatments did not show superiority over surufatinib monotherapy (*P* = 0.133).

Subgroup analysis revealed that surufatinib significantly improved PFS when used as a first-line treatment compared to its use in subsequent lines. First-line surufatinib showed superior mPFS (12.0 months) compared to second-line (9.0 months) and later-line treatments (7.0 months). First-line surufatinib also showed a better ORR (8.6%) and DCR (100%) compared to posterior line therapy. While the improved outcomes observed with surufatinib in the first-line setting are encouraging, they do not alone justify its use as the optimal first-line treatment. We emphasize the need for further studies, including randomized controlled trials and investigations into treatment sequencing, to determine the most appropriate positioning of surufatinib in the therapeutic landscape. On the one hand, in addition to the direct tumor cell killing effect, radiotherapy can also activate the tumor immune microenvironment by promoting the release of a variety of immunomodulatory proteins and inflammatory factors, so that it can change from the immunosuppressive “cold” tumor state to the immune activated “hot” tumor state.^21-24^

In our study, AEs observed in patients receiving surufatinib were consistent with previous studies, with no new safety signals identified. The overall AEs incidence in our study was 43.8% (39/89), with grade 3 or higher AEs at 11.2% (10/89). The majority of treatment-related AEs are mild to moderate, with the most common reactions being hypertension (*n* = 17, 19.1%), diarrhea (*n* = 14, 15.7%), proteinuria (*n* = 12, 13.5%) and hypothyroidism (*n* = 5, 5.6%). Among the 39 cases with surufatinib-related AEs, PR was achieved in 4 cases, while SD was observed in 30 cases. Additionally, the mPFS for these patients was found to be 10.0 months. These indicated that the presence of adverse reactions does not have a substantial impact on the effectiveness of surufatinib. Previous studies have suggested that surufatinib-induced hypertension may serve as a predictive factor for better treatment outcomes; however, further research is required to comprehend the underlying mechanisms behind this phenomenon.^25,26^ AEs associated with surufatinib primarily revolve around anti-angiogenic reactions and current clinical practices have gained extensive experience in effectively managing such reactions to anti-angiogenic drugs proficiently. Furthermore, most AEs can be controlled through dose interruptions and adjustments, confirming the favorable safety profile of surufatinib in treating NET.

We also found that the incidence of AEs associated with surufatinib increases with higher dosages. The incidence of AEs in patients receiving treatment with 300 mg/day and 250 mg/day surufatinib was 61.1% (11/18) and 60% (3/5), respectively, which is significantly higher than those receiving treatment with 200 mg/day surufatinib (36.4%, 22/66). Among patients treated with 300 mg/day surufatinib, 33.3% (6/18) had to reduce their dosage to either 200 mg/day or 250 mg/day due to AE intolerance, resulting in gradual disappearance of adverse reaction symptoms. Additionally, when the treatment dosages for surufatinib were respectively set at 200 mg, 250 mg, and 300 mg, the mPFS was 9.0 months, 14.0 months, and 11.0 months, respectively. In conclusion, the occurrence rate of AEs in the oral surufatinib 200 mg/day cohort exhibited a significant reduction as compared to the high-dose group, while preserving similar effectiveness. When devising an appropriate dosage schedule, it is imperative to carefully evaluate the comprehensive risk-benefit profile. Our findings suggest that a daily dosage of 200 mg may be deemed appropriate for oral administration of surufatinib, with the possibility of considering additional doses if well-tolerated.

As a retrospective study, this research conducted the most comprehensive single-center analysis of the efficacy and safety of surufatinib in TNETs. However, it is important to acknowledge certain limitations. First, all the patients with TNETs included in our review were exclusively from China, thereby excluding patients from other regions and ethnic backgrounds. Second, due to the rarity and heterogeneity of TNETs, establishing standardized treatment strategies for this population poses significant challenges. Further studies should encompass multicenter and large-scale prospective research to provide more precise insights into the efficacy of surufatinib in treating TNETs.

## Data Availability

All data generated or analyzed during the study are included in this published article. All the authors retain the raw data and could share them with valid reasons, such as inquiries from readers or academic reports.

## Data availability

The data underlying this article will be shared on reasonable request to the corresponding author.
