# Correction to:Efficacy and safety of surufatinib in the treatment of thymic neuroendocrine tumors: a 102-case retrospective study

**DOI:** 10.1093/oncolo/oyaf245

**Published:** 2025-09-17

This is a correction to: Xuebing Jia, Zhichen Sun, Junyan Xu, Dan Huang, Fengzhen Chen, Jie Chen, Yun Liang, Efficacy and safety of surufatinib in the treatment of thymic neuroendocrine tumors: a 102-case retrospective study, *The Oncologist*, Volume 30, Issue 5, May 2025, https://doi.org/10.1093/oncolo/oyaf072

In the originally published version of the manuscript, there were several errors - these do not affect the key conclusions or validity of the study.

In Results section, under subheading Adverse Events, paragraph 2, text is emended to read: text is emended to read: “[…]including 4 cases of grade 3 or higher diarrhea (22.2%)[…]” instead of “[…]including 4 cases of grade 3 or higher diarrhea (22.2%)[…]”. In the same paragraph, to align with the revised cohort size, text is emended to read: “Among 18 patients on 300 mg/day[…]” instead of: “Among 22 patients on 300 mg/day[…]”.

In section Discussion, paragraph 6, second sentence, text is emended to read: “[…]60% (3/5)[…]” instead of: “[…]60% (3/5)[…]”.

The emendations have been made to the article.

